# Relationship Between the Number of Patients Visiting Emergency Department and Tokyo Health System’s Capacity During Early Stages of the First Wave of COVID-19

**DOI:** 10.1007/s42399-020-00583-8

**Published:** 2020-10-12

**Authors:** Yohei Ishikawa, Toru Hifumi, Norio Otani, Ryosuke Miyamichi, Mitsuyoshi Urashima, Satoshi Takeda, Shinichi Ishimatsu

**Affiliations:** 1 Department of Emergency and Critical Care Medicine, St. Luke’s International Hospital, 9-1 Akashi-cho, Chuo-ku, Tokyo 104-8560 Japan; 2 Division of Molecular Epidemiology, Jikei University School of Medicine, Tokyo, Japan; 3 Department of Emergency Medicine, Jikei University School of Medicine, Tokyo, Japan

**Keywords:** COVID-19, SARS-CoV-2, Japanese Ministry of Health, Labour and Welfare, Capacity

## Abstract

The first coronavirus 2019 (COVID-19) patients were reported in China on December 12, 2019, and the first COVID-19 patients were reported in Japan on January 16, 2020. Here, we investigated the number of patients in Emergency Departments (EDs) in three major hospitals in Tokyo, and also briefly discussed about the relationship between the number of patients in EDs and health system’s capacity. We compared the number of patients in 2020 to the average number of patients from 2016 to 2019. Numbers were compared in three periods: before the first COVID-19 patient was reported in Japan (January 1 to January 16), after the government encouraged social distancing (February 26 to March 10), and the interval between them (January 17 to February 25). The average number of daily patients in 2020 (*n* = 122) decreased by 17% compared to the average number of patients from 2016 to 2019 (*n* = 144) (Mann-Whitney test, *p* < 0.001). This phenomenon might be due to a fear of contracting the virus at hospitals, companies having their employees work remotely and postponing events, people following the Japanese Ministry of Health, Labour and Welfare’s instructional guidelines for going to the hospital, prevention awareness becoming widespread, and a decreased number of tourists. The number of patients visiting Emergency Departments in Tokyo was decreased and the number of COVID-19 infections has remained within the health system’s capacity during the early phase of COVID-19 first wave.

## Introduction

The first coronavirus 2019 (COVID-19) patients were reported in China on December 12, 2019, and the first COVID-19 patients were reported in Japan on January 16, 2020. The total number of cases in Japan had increased to 552 patients (including 12 deaths) by March 10 [1]. The Japanese Ministry of Health, Labour and Welfare’s (MHLW) response to COVID-19 included giving instructions on when to go to the hospital (2 days after having a fever or other symptoms for high-risk people, and 4 days after having a fever or other symptoms for normal-risk people), setting up official call centers for patients with suspected COVID-19 and adjusting hospitals to accept them [2], encouraging companies to have employees work remotely, recommending the cancellation of events [1], advising the closure of all elementary and junior high schools in the country [3], and requiring everyone arriving from the affected area by sea and air travel to be isolated for 14 days at a location designated by the quarantine station chief and not use public transportation in Japan, whether symptomatic or asymptomatic [1].

Initially, staff in Emergency Departments (EDs) anticipated the number of patients to increase. In actuality, the number of patients seemed to decrease.

Here, we investigated the number of patients in EDs in three major hospitals in Tokyo, and also briefly discussed about the relationship between the number of patients in EDs and health system’s capacity.

## Methods

Hospital characteristics (total number of beds, critical care medical center) and the average number of patients visiting EDs per day in three hospitals (The Jikei University Hospital, The Jikei University Daisan Hospital, and St. Luke’s International Hospital) were collected. We compared the number of patients in 2020 to the average number of patients from 2016 to 2019 in three major hospitals in Tokyo. Numbers were compared in three periods: before the first COVID-19 patient was reported in Japan (January 1 to January 16), after the government encouraged social distancing (February 26 to March 10), and the interval between them (January 17 to February 25). The Mann-Whitney test was used to perform the comparison of the number of daily patients in 2020 and the 2016–2019 average in 3 Tokyo EDs.

## Results and Discussion

**Fig. 1:**
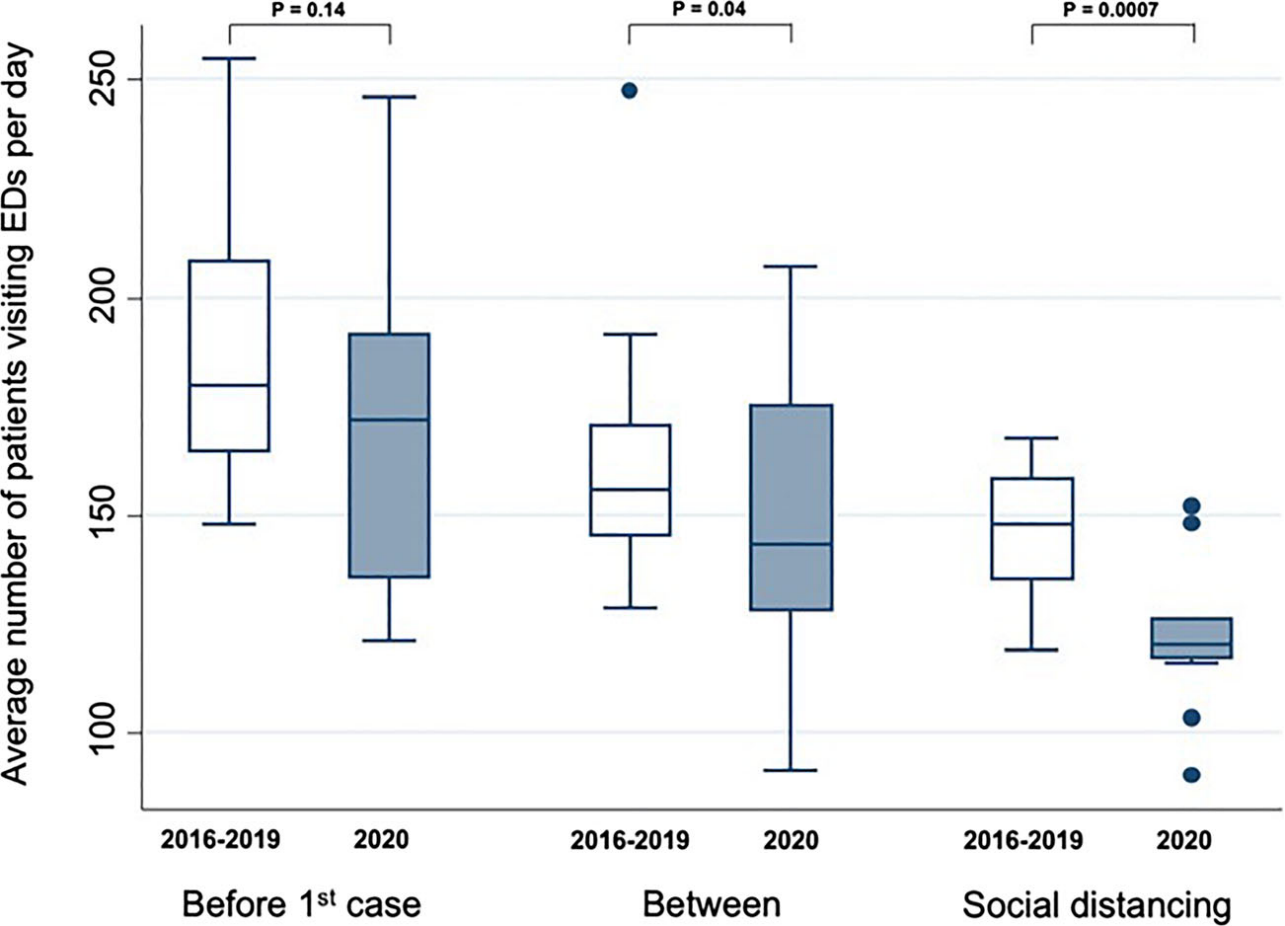
Comparison of the number of daily patients in 2020 and the 2016–2019 average in 3 Tokyo Emergency Departments (EDs). “Before 1^st^ case” denotes the period of January 1st to 16th, “Between” denotes the period of January 17th to February 25th, and “Social distancing” denotes the period of February 26th to March 10th. The Mann-Whitney test was used

Characteristics and the average number of patients visiting EDs per day in three hospitals are showed in Table 1. Our results show, especially after the government announced social distancing on February 25, that the average number of daily patients in 2020 (*n* = 122) decreased by 17% compared to the average number of patients from 2016 to 2019 (*n* = 144) (Mann-Whitney test, *p* < 0.001; Fig. 1).

**Table 1:** Characteristics and the average number of patients visiting Emergency Department per day in three hospitals

	The Jikei University Hospital	The Jikei University Daisan Hospital	St. Luke’s International Hospital
Total number of beds	1075	581	520
Critical care medical center	No	No	Yes
The number of patients before the first COVID-19 patient was reported in Japan (January 1 to January 16)			
2016	31.5	50.7	85.6
2017	34.3	54.8	92.6
2018	39.3	61.0	96.4
2019	38.6	60.1	106.2
2020	35.9	41.6	90.6
The number of patients (January 17 to February 25)			
2016	31.6	41.1	88.7
2017	30.2	39.6	78.8
2018	37.1	41.2	87.4
2019	32.2	42.0	88.3
2020	31.3	34.5	82.5
The number of patients after the government encouraged social distancing (February 26 to March 10)			
2016	33.9	40.9	81.0
2017	31.6	34.5	69.6
2018	31.8	33.5	79.2
2019	32.5	34.2	82.6
2020	22.7	28.1	70.9

This phenomenon might be due to a fear of contracting the virus at hospitals, companies having their employees work remotely and postponing events, people following the MHLW instructional guidelines for going to the hospital, prevention awareness becoming widespread, and a decreased number of tourists.

Decrease in number of patients visiting EDs during COVID-19 pandemic has been also reported in several countries possibly due to lockdown and fear of contagion [4–6]; however, lockdown restrictions have not initiated in Tokyo [7]. Moreover, consideration of universal health insurance system in Japan, it was highly expected that many patients would visit the EDs for obtaining peace of mind as well as physical health. Thus, it is worth noting that same tendency was observed in Japan with such unique situations.

Although several discussions have occurred with regard to the instructions (i.e. patients should contact consultation offices if they had flulike symptoms and a fever over 37.5 °C that continued for four days) proposed by MHLW, this contributed the decrease in number of patients visiting EDs and maintain the health system’s capacity in the early stage of the COVID-19 first wave.

## Conclusions

The number of patients visiting Emergency Departments in Tokyo was decreased and the number of COVID-19 infections has remained within the health system’s capacity during the early phase of COVID-19 first wave.
